# The molecular epidemiology of *Stenotrophomonas maltophilia *bacteraemia in a tertiary referral hospital in the United Arab Emirates 2000–2004

**DOI:** 10.1186/1476-0711-5-32

**Published:** 2006-12-28

**Authors:** Pauline A Jumaa, Agnes Sonnevend, Tibor Pàl, Mohammed El Hag, Ray Amith, Omar Trad

**Affiliations:** 1 Department of Medical Microbiology, Faculty of Medicine and Health Sciences, UAE University, Al Ain, United Arab Emirates; 2 Department of Microbiology, Tawam Hospital, P O Box 15258, Al Ain, United Arab Emirates; 3 Department of Paediatric Oncology, Tawam Hospital, P O Box 15258, Al Ain, United Arab Emirates; 4 Department of Clinical Microbiology and Infection Control, University Hospital Birmingham NHS Foundation Trust, Queen Elizabeth Hospital, Edgbaston, Birmingham, B15 2TH, UK

## Abstract

**Background:**

*Stenotrophomonas maltophilia *is recognised as an important cause of nosocomial infection, especially in immunocompromised patients, resulting in significant morbidity and mortality. The treatment of *S. maltophilia *infection presents a therapeutic challenge. The precise modes of transmission of *S. maltophilia *in the hospital environment are not known and such knowledge is essential to target interventions to prevent spread. There are few published data on the patterns of nosocomial infection in the United Arab Emirates (UAE). A recent study showed that *S. maltophilia *is an established cause of bloodstream infection in Tawam Hospital in the UAE. Little is known about its epidemiology in the hospital.

**Methods:**

We describe the clinical characteristics of 25 episodes of *S. maltophilia *bacteraemia which occurred from 2000–2004. The strains were characterised using pulsed field gel electrophoresis (PFGE).

**Results:**

All episodes were hospital-acquired and malignancy and central venous catheters were predisposing factors. Catheter-associated infection comprised 88% infection. Catheter removal was important for the successful management of catheter-associated infection. The results of PFGE suggested that there were as many strains as patients. *S. maltophilia *strains isolated from the same patient had indistinguishable PFGE profiles.

**Conclusion:**

PFGE is a valid and reproducible typing method for *S. maltophilia*. The precise sources and modes of spread of *S. maltophilia *in the hospital are still not known. Knowledge that person to person transmission was not a major mode of transmission enabled infection control interventions for *S. maltophilia *to be targeted more effectively.

## Background

*Stenotrophomonas maltophilia *is a ubiquitous pathogen and has been isolated from a wide variety of sources, mainly water-associated, both inside and outside the hospital environment [1,2].

*S. maltophilia *is now recognised as an important cause of hospital-acquired infection, causing significant morbidity and mortality in immunocompromised patients [1,3]. Colonisation and infection with *S. maltophilia *is associated with mechanical ventilation, the presence of a central venous catheter (CVC), neutropenia, cytotoxic chemotherapy [1]. In most cases, isolation of *S. maltophilia *from clinical specimens from non-sterile sites represents colonisation rather than infection and *S. maltophilia *is considered a low-virulence pathogen. Bloodstream infection with *S. maltophilia *is one of the least subjective diagnoses to make based on the isolation of *S. maltophilia *from blood cultures associated with signs and symptoms of infection. In contrast, the isolation of *S. maltophilia *from respiratory secretions, from which other organisms are also isolated, may represent colonisation and another pathogen may be responsible for the clinical signs and symptoms of pneumonia rather than *S. maltophilia*. However, *S. maltophilia *may cause serious infections such as bacteraemia, pneumonia, meningitis, skin and soft tissue infection and there is evidence that its incidence is increasing [1]. Optimizing the antimicrobial treatment of *S. maltophilia *infection is challenging because *S. maltophilia *is inherently resistant to many antimicrobials [3]. Also, routine antimicrobial susceptibility tests performed using disc diffusion tests are often unreliable for *S. maltophilia *[2,11].

Despite being recognised as a nosocomial pathogen, the precise modes of transmission of *S. maltophilia *in the hospital environment are not known. Molecular characterisation of microorganisms can be used to provide evidence of epidemiological relationships between strains and is an important tool in the investigation of the spread of infectious diseases [4]. Such molecular fingerprinting methods to compare strains can provide useful information about patterns of infection with *S. maltophilia *and possible sources and modes of transmission [5,6]. Pulsed-field gel electrophoresis (PFGE) has been established as a discriminatory method for typing *S. maltophilia *strains [5,6]. Recent studies have compared other typing methods such as ribotyping and ERIC-PCR with PFGE [7,8]. While these other methods may have advantages over PFGE in terms of rapidity, PFGE has been shown in studies to be discriminatory [7,8]. PFGE was available in our laboratory.

There are few data on the patterns of nosocomial infection in the United Arab Emirates (UAE). A literature search using PubMed did not identify any reports on *S. maltophilia *from the UAE. However, *S. maltophilia *is an established pathogen in Tawam Hospital, which is a major tertiary referral hospital in the UAE. In a recent study of paediatric oncology blood culture isolates in Tawam hospital, 3% of Gram-negative isolates were *S. maltophilia *[9]. Nothing else is known about the epidemiology of *S. maltophilia *in the hospital or whether the strains represent a single clone, suggesting a common source or person to person spread. We report the clinical characteristics of bacteraemia in Tawam Hospital, UAE and investigate the molecular epidemiology of *S. maltophilia *bacteraemia isolates from the hospital aiming to identify targets to prevent spread.

## Methods

### Setting

Tawam Hospital is a 400 bed tertiary care and general hospital in Al Ain, UAE and is a major cancer referral centre for the UAE and the Gulf region. It is also a regional centre for neurosurgery. Specialist units include intensive care, renal dialysis, neonatal intensive care, neurosurgery, adult and paediatric oncology.

### Surveillance methods

Clinical and microbiology data were collected from cases of invasive *S. maltophilia *infection. Clinical data included: age; clinical department; underlying disease; whether the infection was hospital-acquired; origin of bacteraemia; presence of a central venous catheter; whether the patient was immunocompromised; antimicrobial therapy; outcome. Microbiology data included: whether *S. maltophilia *was isolated in pure culture; the results of susceptibility tests using E-tests (ABbiodisk, Solna, Sweden).

### Definitions

Hospital-acquired bloodstream infection was defined according to accepted criteria [10]. Polymicrobial was defined as more than 1 organism isolated from the same episode which yielded the *S. maltophilia*. Outcome was defined as death within 7 days of *S. maltophilia *bacteraemia.

### Microbiology methods

Blood cultures were analysed using the Vital analyzer (bioMerieux, Marcy-l'Etoile, France) from 2000–2002 and Bactec 9240 (BD Diagnostics, USA) from 2003. Blood cultures were incubated routinely for 7 days. Positive blood cultures and other clinical specimens were investigated using routine culture methods. Suspect colonies were identified as *S. maltophilia *using the API 20 NE system (bioMerieux, Marcy-l'Etoile, France). Strains of *S. maltophilia *were saved on nutrient agar slopes wherever possible. All strains were tested using E-tests (ABbiodisk, Solna, Sweden) for susceptibility to cotrimoxazole and meropenem. Meropenem resistance was used to help to confirm the identification of *S. maltophilia *isolates. Only susceptibility to cotrimoxazole was reported in accordance with available guidelines from the National Committee for Clinical Laboratory Standards (NCCLS) [11].

### Data storage and analysis

Data were stored and analysed in Microsoft Excel.

### Molecular typing by Pulsed Field Gel Electrophoresis (PFGE)

The pulsed field gel electrophoresis technique used was based on the method published by Denton et al with modifications [6]. Overnight cultures of *S. maltophilia *on Blood Agar Base (Oxoid, Basingstoke, UK) were suspended into SE buffer (25 mM Na-EDTA pH 8.0, 75 mM NaCl), and the optical density at 600 nm was adjusted to 1.6–1.7. Plugs were prepared by mixing 300 μl of the bacterial suspensions with 700 μl 1.0% plug agarose (Sigma-Aldrich, St. Louis, MO). Cell lysis was carried out in two steps. First the plugs were incubated for 5 hours at 37°C in 1 ml lysis buffer (10 mM TRIS-HCl pH 8.0, 100 mM Na-EDTA pH 8.0, 50 mM NaCl, 0.2% Na-deoxycholate, 1% sarcosyl, 30 μg/ml RNase A, 2 mg/ml lysozyme), than the lysis buffer was then replaced by 500 μl of proteinase K buffer (100 mM Na-EDTA pH 8.0, 0.4% Na-deoxycholate, 1% sarcosyl) containing 1 mg/ml proteinase K (GibcoBRL) and incubated overnight at 56°C. Plugs were washed 6 times for 20 minutes in TE buffer (10 mM TRIS-HCl pH 8.0, 1 mM Na-EDTA pH 8.0) than stored in TE buffer at 4°C.

Prior digestion plugs were placed into 100 μl of restriction buffer 2 (NEBiolabs, Beverly, USA) for 30 minutes. This buffer was subsequently replaced with 100 μl fresh restriction buffer 2 containing 30 U Xba I (NEBiolabs, Beverly, USA) and incubated overnight at 37°C. Electrophoresis was performed in 1.2% agarose (Sigma-Aldrich, St. Louis, MO) on a CHEF DRII apparatus (BioRad, Hercules, CA) with 6 V/cm for 22 hours at 14°C with an initial switch time of 5 seconds and a final switch time 35 seconds with linear ramp. Lambda ladder (NEBiolabs, Beverly, USA) was included at the two side lanes of every gel as molecular weight marker. The gels were stained with ethidium bromide, photographed and stored electronically for analysis. The macrorestriction patterns of the strains were compared according to Dice similarity index (1% tolerance interval) using the GelCompare II software (Applied Maths, Sint-Martens-Latem, Belgium). A PFGE cluster was arbitrarily defined as strains showing more than 90% similarities in banding patterns.

## Results

From 2000–2003, 27 episodes of *S. maltophilia *bacteraemia were identified. In 2 cases, clinical and laboratory findings suggested that the isolates did not represent true infection and were contaminants. These were excluded from the analysis.

The clinical findings are summarised in Table 1. Table 2 shows the patients' details. Table 3 shows the other organisms isolated with *S. maltophilia *in episodes of polymicrobial bacteraemia.

**Table 1:** Clinical characteristics of *Stenotrophomonas maltophilia *bacteraemia Tawam Hospital 2000–2004

	**Number (n = 25)**	**(%)**
**Age**		
Adult	18	(72.0)
Child	7	(28.0)
**Hospital Acquired**	25	(100)
**Clinical Unit**		
Adult Oncology	11	(44.0)
Paediatric Oncology	5	(20.0)
Intensive Care Unit^+^	3	(12.0)
Renal Dialysis Unit	4	(16.0)
Neonatal Intensive Care	1	(4.0)
Paediatric Medical	1	(4.0)
**Underlying Disease**		
Malignancy	19	(76.0)
End stage renal failure	4	(16.0)
Prematurity	1	(4.0)
End stage respiratory failure	1	(4.0)
**Immunocompromised**	25	(100)
**Source**		
Line-associated	22	(88.0)
Febrile neutropenia	1	(4.0)
Pneumonia	1	(4.0)
Skin soft tissue infection	1	(4.0)
**Polymicrobial**	9**	(36.0)
**Treatment of line infection**		
Line removal	11/12*	(91.7)
**Antimicrobial therapy**		
Cotrimoxazole	14/16*	(87.5)
**Outcome**		
Death	2	
Death attributed to *S. maltophilia*	0	

^+ ^All were adult oncology patients

* Where data were available ** Isolated with 1–3 other organisms

**Table 2:** Patient details for cases of *Stenotrophomonas maltophilia *bacteraemia

**Patient**	**Gender**	**Age**	**Underlying disease**	**Source**	**Neutropenic**	**Outcome**	**Death attributed to *S. maltophilia *bacteraemia**
1	F	9 years	ALL	Line	Y	Recovered	NA
2	F	5 years	Neuroblastoma	Line	Y	Recovered	NA
3	M	59 years	End Stage Renal Failure	Line	N	Recovered	NA
4	M	52 years	End Stage Renal Failure	Line	N	Recovered	NA
5	F	54 years	End Stage renal failure	Line	N	Recovered	NA
6	M	15 months	Bronchopulmonary dysplasia	Line	N	Recovered	NA
7	M	30 years	ALL	Line	Y	Died	No*
8	M	18 years	ALL	Cellulitis	Y	Recovered	NA
9	M	25 years	T-cell NHL	Pneumonia	N	Died	No*
10	M	40 years	AML	Line	N	Recovered	NA
11	M	56 years	AML	Line	N	Recovered	NA
12	M	28 years	Bowel carcinoma	Febrile neutropenia	Y	Recovered	NA
13	M	17 days	Prematurity	Line-related abscess	N	Recovered	NA
14	F	27 years	Osteosarcoma	Line	Y	Recovered	NA
15	M	48 years	NHL	Line	N	Recovered	NA
16	F	46 years	Breast carcinoma	Line	N	Recovered	NA
17	M	10 years	ALL	Line	N	Recovered	NA
18	F	22 years	ALL	Line	N	Recovered	NA
19	F	35 years	AML	Line	N	Recovered	NA
20	F	6 years	ALL	Line	N	Recovered	NA
21	F	49 years	End Stage Renal Failure	Line	N	Recovered	NA
22	M	32 years	AML	Line	Y	Recovered	NA
23	M	4 years	ALL	Line	N	Recovered	NA
24	F	18 years	ALL	Line	N	Recovered	NA
25	M	41 years	NHL	Line	Y	Recovered	NA

NA = Not applicable; ALL = Acute lymphoblastic leukaemia; AML = Acute myeloid leukaemia; NHL = Non-Hodgkins Lymphoma

* Death attributed to invasive aspergillosis

**Table 3:** Organisms isolated with *Stenotrophomonas maltophilia *in polymicrobial bacteraemia episodes

**Organism**	**Number of episodes**
*Pseudomonas aeruginosa*	3
*Acinetobacter *sp	3
Coagulase-negative staphylococcus	2
*Klebsiella *sp	2
*Enterobacter *sp	2
*Bacillus *sp	1
*Kluyvera *sp	1

All *S. maltophilia *isolates were susceptible to cotrimoxazole with minimum inhibitory concentrations (MIC) less than 0.5 mg/L, and a range of 0.016–0.25 mg/L. All strains tested were resistant to meropenem with MIC's >32.0 mg/L.

**Figure 1:**
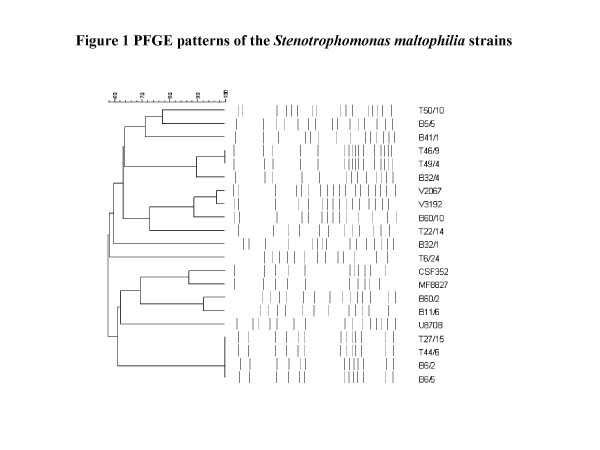
**Pulsed field gel electrophoresis (PFGE) patterns of the *Stenotrophomonas maltophilia *strains**. T 46/9 and T 49/4 are isolates from patient 21 on Table 2. T 27/15, T 44/6, B6/2, B6/5 are isolates from patient 17 on Table 2. V3192 and V2067 represent patients 6 and 9 respectively on Table 2.

There were 21 strains available for PFGE analysis. Figure 1 shows the PFGE patterns. Of these, 16 distinct patterns, including three clusters of patterns with over 95% similarity were detected. Two of the clusters, incorporating strains T46/9 and T49/4 and strains T27/15, T44/6, B6/2, B6/5 respectively, were isolated from the same patients. The latter group was isolated over a period spanning 8 months. The third cluster (V2067 and V3192) were two strains from two different patients on different wards at different times. Duplicate strains from the same patient showed indistinguishable PFGE profiles (data not shown). Overall, *S. maltophilia *isolates in our hospital represented diverse strains and they were unrelated. With the exception of the three clusters, 2 of which comprised isolates from the same patients, there were as many PFGE types as patients.

## Discussion

The major aim of our study was to try to investigate the epidemiology of *S. maltophilia *isolates in our hospital and thus identify targets to attempt to interrupt its spread in the hospital environment. While there have been larger, more detailed studies reported in the medical literature, this is the first such report from the UAE and therefore we do not know whether our experience is typical of the UAE.

Although we did not compare our population with matched controls, the clinical characteristics with *S. maltophilia *bacteraemia in our hospital in the UAE are broadly similar to those reported worldwide. All *S. maltophilia *bacteraemia episodes were hospital-acquired and malignancy and central venous catheters were major predisposing factors. Line removal seemed important in the successful management of line-associated infection, though the numbers investigated were small. Of the 22 cases where the clinical and microbiological features suggested that line infection was likely, in only 12 cases was it clearly documented in the clinical records that line removal had taken place.

We found that *S. maltophilia *bacteraemia was polymicrobial in 9 out of 25 episodes. This characteristic has been noted some studies, but not in others and the frequency with which *S. maltophilia *was isolated from mixed cultures in earlier studies led to delay in recognising its pathogenic potential [1,12]. It is not possible to be absolutely sure if *S. maltophilia *isolated from polymicrobial episodes represented a true pathogen, since the other organisms isolated from polymicrobial episodes in our patients were also well-recognised pathogens. However, in those cases were *S. maltophilia *was the sole isolate, the clinical features suggested that *S. maltophilia *was behaving as a pathogen.

There were only two deaths in our series of patients and both of these were attributed to causes other than *S. maltophilia *bacteraemia. We measured mortality at 7 days and it is possible that the mortality would have been higher if we had used 30 day – mortality. Establishing the mortality of *S. maltophilia *bacteraemia from the literature is difficult because different studies have used different mortality definitions [13,14].

The results of the PFGE suggest that person to person spread was not the major mode of transmission of *S. maltophilia *in this hospital. We found repeat that duplicate isolates of *S. maltophilia *had indistinguishable PFGE profiles, supporting the validity and reproducibility of PFGE as a fingerprinting method. One patient had 3 separate episodes of *S. maltophilia *bacteraemia associated with portacath infections, spanning 8 months. PFGE of 4 strains from these 3 episodes revealed indistinguishable profiles, suggesting that the same strain may have been acquired from the same unidentified environmental reservoir. However, further investigations are necessary to support this observation.

We did not find the susceptibility tests performed helpful in distinguishing the strains or in choosing antimicrobial therapy. Routine susceptibility tests are unreliable for testing *S. maltophilia *[11]. We recommended cotrimoxazole for antimicrobial therapy of *S. maltophilia *infection as it is considered the antimicrobial of choice for *S. maltophilia *infection. All the strains tested were susceptible using the breakpoint recommended by NCCLS guidelines [11].

In previous studies *S. maltophilia *has been isolated from a wide variety of hospital sources [1]. We suggest that *S. maltophilia *isolates in this hospital originate from numerous sources in the hospital environment. We were able to direct our attention to possible environmental sources, such as improving the compliance with infection control procedures involved in central venous catheter care rather than interventions to prevent person to person spread, such as single room isolation. Further work is necessary to identify these sources.

## Conclusion

Our study highlights that PFGE and other similar typing schemes are essential to investigate relationships between isolates and therefore to provide information to identify targets and strategies to control the spread of nosocomial pathogens. The sources and precise modes of spread of *S. maltophilia *in our hospital are still not known. However, person to person transmission of *S. maltophilia *seems a rare occurrence and knowledge of this is important in enabling scarce infection control resources to be targeted most appropriately.

## Competing interests

The author(s) declare that they have no competing interests.

## Authors' contributions

PJ conceived of the study, participated in its coordination and design and drafted the manuscript

AS participated in its design and the molecular analysis

TP participated in its coordination and design and molecular analysis

MH Participated in the molecular analysis and the antimicrobial susceptibility testing

RA participated in the coordination of the microbiological and biochemical analysis

OT participated in the collection, analysis and interpretation of the clinical data

All authors have read and approved the final manuscript

## References

[B1] Denton M, Kerr KG (1998). Microbiological and clinical aspects of infection associated with *Stenotrophomonas maltophilia *infection. Clin Microbiol Rev.

[B2] Gilligan PH, Whittier S, Murray PR, Baron EJ, Pfaller MA, Tenover FC, Yolken RH (1999). *Burkholderia Stenotrophomonas*, *Ralstonia*, *Brevundimonas*, *Comomonas *and *Acidovorax*. Manual of Clinical Microbiology.

[B3] Gales AC, Jones RN, Forward KR, Linares J, Sader HS, Vernhoef J (2001). Emerging importance of multidrug-resistant *Acinetobacter *species and *Stenotrophomonas maltophilia *as pathogens in seriously ill patients: geographic patterns, epidemiological features and trends in the SENTRY antimicrobial surveillance programme (1997–1999). Clin Infect Dis.

[B4] Pfaller MA, Acar J, Jones RN, Verhoef J, Turnidge J, Sader HS (2001). Integration of molecular characterization of microorganisms in a global antimicrobial resistance surveillance program. Clin Infect Dis.

[B5] Berg G, Roskot N, Smalla K (1999). Genotypic and phenotypic relationships between clinical and environmental isolates of *Stenotrophomonas maltophilia*. J Clin Microbiol.

[B6] Denton M, Todd N, Kerr K, Hawkey P, Littlewood J (1998). Molecular epidemiology of *Stenotrophomonas maltophilia *isolated from clinical specimens from cystic fibrosis patients and associated environmental samples. J Clin Microbiol.

[B7] Gulmez D, Hascelik G (2005). *Stenotrophomonas maltophilia*: antimicrobial resistance and molecular typing of an emerging pathogen in a Turkish University Hospital. Clin Microbiol Infect.

[B8] Silbert S, Pfaller MA, Hollis RJ, Barth AL, Sader HS (2004). Evaluation of three molecular typing techniques for nonfermentative Gram-negative bacilli. Infect Control Hosp Epidemiol.

[B9] Trad O, Jumaa PA, Afify Z (2003). The changing pattern of bloodstream infection in pediatric oncology patients in the United Arab Emirates. Pediatr Hematol Oncol.

[B10] Garner JS, Jarvis WR, Emori TG, Horan TC, Hughes JM (1998). CDC definitions for nosocomial infections 1998. Am J Infect Control.

[B11] Clinical and Laboratory Standards Institute/NCCLS (2005). Performance Standards for Antimicrobial Susceptibility Testing: Fifteenth Informational Supplement. CLSI/NCCLS document M100-S15. Clinical and Laboratory Standards Institute, Wayne, Pennsylvania, USA.

[B12] Jang T-N, Wang FD, Wang LH, Liu CY, Liu LM (1992). *Xanthomonas maltophilia *bacteremia: an analysis of 32 cases. J Formos Med Assoc.

[B13] Muder RR, Harris AP, Muller S, Edmond M, Chow JW, Papadakis K, Wagener MW, Bodey GP, Steckelberg JM (1996). Bacteremia due to *Stenotrophomonas (Xanthomonas) maltophilia*: a prospective, multicenter study of 91 episodes. Clin Infect Dis.

[B14] Friedman ND, Korman TM, Fairley CK, Franklin JC, Spelman DW (2002). Bacteraemia due to *Stenotrophomonas maltophilia*: an analysis of 45 episodes. J Infect.

